# Editorial: New Insights on Sialic Acid and Sialylated Glycans in the Tumorigenic Process

**DOI:** 10.3389/fonc.2026.1795897

**Published:** 2026-06-09

**Authors:** Rachel A. Willand-Charnley

**Affiliations:** South Dakota State University, Brookings, SD, United States

**Keywords:** aberrant glycosylation, glycobiology of cancer, polysialic acid, sialic acid, tumorgenesis

Aberrant glycosylation is a defining feature of many cancers and plays an essential role in tumor initiation, progression, and metastasis. Among the many glycan alterations associated with malignant transformation, changes in the expression and structure of sialic acids and sialylated glycans have emerged as critical determinants of tumor behavior. Sialic acids are a family of nine-carbon acidic monosaccharides typically located at the terminal positions of glycans on glycoproteins and glycolipids at the cell surface. Their prominent position and negative charge allow them to regulate key biological processes including cell-cell communication, receptor signaling, immune recognition, and host-pathogen interactions. In cancer, dysregulated sialylation frequently leads to the accumulation of highly sialylated glycoconjugates on tumor cell surfaces, which can influence tumor growth, metastatic potential, and immune escape.

The hypersialylation commonly observed in tumors is often driven by altered expression or activity of glycosylation enzymes, particularly sialyltransferases and sialidases. These enzymatic changes remodel the tumor glycocalyx, the dense glycan layer surrounding the cell surface, thereby modifying the physical and biochemical properties of cancer cells. Increased sialylation can enhance tumor cell motility, promote adhesion to endothelial cells, and facilitate metastatic dissemination. At the same time, sialylated glycans can interact with inhibitory immune receptors, most notably members of the sialic acid–binding immunoglobulin-like lectin (Siglec) family, leading to suppression of anti-tumor immune responses. Because of these diverse functions, sialylation has gain recognition as a key molecular interface between tumor cells and the immune system.

Recent advances in glycobiology, chemical biology, and analytical technologies have significantly expanded our ability to investigate glycan structures and functions in cancer. Improved glycomics and glycoproteomics tools now allow detailed characterization of tumor-associated glycans and their dynamic regulation. In parallel, genetic and pharmacological approaches to investigate glycosylation pathways are providing new insights into the functional roles of sialylated glycans in tumorigenesis. These advances are also opening new opportunities for translational applications, including the development of glycan-based biomarkers and therapeutic strategies that target sialylation pathways.

The Research Topic “New Insights on Sialic Acid and Sialylated Glycans in the Tumorigenic Process” brings together seven contributions that collectively highlight emerging roles for sialic acids in cancer biology. The articles included in this Research Topic encompass original research and review articles addressing tumor-immune interactions, glycan structural modifications, microenvironmental influences on sialylation, and translational applications of tumor-associated glycans. Together, these studies provide new perspectives on how sialylated glycans regulate tumor progression and illustrate potential avenues for therapeutic intervention.

The central theme emerging from several contributions is the role of sialylated glycans in regulating anti-tumor immunity. In the article *Polysialic acid is upregulated on activated immune cells and negatively regulates anticancer immune activity*, the authors demonstrate that the carbohydrate polymer polysialic acid (polySia) is markedly upregulated on immune cells within the breast tumor microenvironment. Their study reveals that activation of primary T cells and macrophages induces polySia expression, which is attached to multiple carrier proteins within these cells. Importantly, selective removal of polySia enhanced the ability of innate immune cells to kill breast cancer cells, indicating that polySia functions as a negative regulator of anti-tumor immune responses. These findings highlight how glycan remodeling within immune cells themselves can contribute to immune suppression in the tumor microenvironment.

Complementing this work, the study *CD16 and Siglec expression refine the phenotypic heterogeneity of steady-state myeloid-derived suppressor cells* explores the role of Siglec expression in defining neutrophil subpopulations associated with immunosuppressive activity. Myeloid-derived suppressor cells (MDSCs) represent a heterogeneous group of cells that contribute to immune suppression during cancer progression. By examining the expression of multiple Siglec family members across distinct neutrophil populations, the authors demonstrate differential expression patterns of Siglec-3, Siglec-5/14, Siglec-7, and Siglec-9. Their results further show that combining CD16 and Siglec-9 expression provides improved resolution of neutrophil subsets and suggests that not all low-density neutrophils possess classical MDSC activity. These findings highlight the complexity of Siglec-mediated immune regulation and emphasize the need for more refined markers to distinguish immunosuppressive cell populations in cancer.

Another contribution addressing tumor immune evasion is presented in *Sialylation inhibition improves macrophage mediated tumor cell phagocytosis of breast cancer cells triggered by therapeutic antibodies of different isotypes*. In this study, the authors show that pharmacological inhibition of sialyltransferases using the compound P-3Fax-Neu5Ac reduces cell surface sialylation on breast cancer cells and enhances antibody-dependent cellular phagocytosis by macrophages. Notably, this effect was observed across antibodies of different isotypes and tumor antigens, suggesting that reducing tumor hypersialylation may broadly improve the efficacy of antibody-based cancer therapies. The authors further observed reduced binding of Siglec-7 and Siglec-9 following sialylation inhibition, supporting the idea that sialic acid–Siglec interactions function as immune checkpoints that limit anti-tumor immunity.

Beyond immune regulation, several, investigators explore how the tumor microenvironment influences glycan remodeling. In *Adipose microenvironment promotes hypersialylation of ovarian cancer cells*, the authors demonstrate that adipose tissue-derived factors can drive increased expression of multiple sialyltransferases in ovarian cancer cells. Using transcriptomic and glycan labeling approaches, the study identifies stable subpopulations of cancer cells characterized by high or low levels of sialylation. Interestingly, only the hypersialylated population successfully established tumors in adipose tissue in immunocompetent mouse models, whereas both populations formed tumors in immunodeficient animals. These findings suggest that hypersialylation may provide a mechanism for tumor cells to evade immune surveillance within adipose-rich metastatic niches.

Chemical modifications of sialic acids themselves may also influence cancer progression. The study *Early in vitro results indicate that de-O-acetylated sialic acids increase Selectin binding* in cancers investigates how O-acetylation of sialic acids affects selectin-mediated interactions. Through genetic manipulation of enzymes regulating O-acetylation, the authors demonstrate that increased levels of de-O-acetylated sialic acids enhance selectin binding and promote cell proliferation and migration in lung and colon cancer models. These findings provide new insights into how structural variations in sialic acids can modulate metastatic processes.

The translational potential of tumor-associated glycans is highlighted in *Sialyl-Tn glycan epitope as a target for pancreatic cancer therapies*. This study examines the expression of the truncated O-glycan antigen sialyl-Tn (STn) in pancreatic cancer and demonstrates that STn is strongly expressed in multiple pancreatic carcinoma subtypes while absent from normal pancreatic tissue. Importantly, STn expression was maintained across patient-derived xenograft models, supporting their utility for evaluating STn-targeted therapeutic approaches.

Finally, the review article *When a negative (charge) is not a positive: sialylation and its role in cancer mechanics and progression* provides a broader perspective on the functional consequences of tumor hypersialylation. The authors discuss how increased sialylation reshapes the glycocalyx, affecting membrane tension, integrin clustering, and signaling pathways that promote tumor growth and metastasis. The review also highlights the importance of sialic acid–Siglec interactions in immune evasion and discusses emerging strategies aimed at targeting sialylation pathways for cancer therapy.

Collectively, the articles in this Research Topic illustrate the diverse and complex roles of sialic acids and sialylated glycans in tumor biology. From immune modulation and microenvironmental influences to structural glycan variations and translational applications, these studies underscore the expanding importance of glycobiology in cancer research. Continued integration of glycobiology with immunology, chemical biology, and translational oncology is essential for fully understanding the roles of sialylation in tumorigenesis and for developing innovative therapeutic strategies targeting glycan-mediated mechanisms, which will undoubtedly call upon contributions to be made from our most talented interdisciplinary organic chemists.

